# Comparison of performance of automatic recognizers for stutters in speech trained with event or interval markers

**DOI:** 10.3389/fpsyg.2024.1155285

**Published:** 2024-02-27

**Authors:** Liam Barrett, Kevin Tang, Peter Howell

**Affiliations:** ^1^Department of Experimental Psychology, University College London, London, United Kingdom; ^2^Department of English Language and Linguistics, Institute of English and American Studies, Faculty of Arts and Humanities, Heinrich Heine University Düsseldorf, Düsseldorf, Germany; ^3^Department of Linguistics, University of Florida, Gainesville, FL, United States

**Keywords:** stuttering, speech pathology, automatic speech recognition, machine learning, computational paralinguistics, language diversity, language model, whisper

## Abstract

**Introduction:**

Automatic recognition of stutters (ARS) from speech recordings can facilitate objective assessment and intervention for people who stutter. However, the performance of ARS systems may depend on how the speech data are segmented and labelled for training and testing. This study compared two segmentation methods: event-based, which delimits speech segments by their fluency status, and interval-based, which uses fixed-length segments regardless of fluency.

**Methods:**

Machine learning models were trained and evaluated on interval-based and event-based stuttered speech corpora. The models used acoustic and linguistic features extracted from the speech signal and the transcriptions generated by a state-of-the-art automatic speech recognition system.

**Results:**

The results showed that event-based segmentation led to better ARS performance than interval-based segmentation, as measured by the area under the curve (AUC) of the receiver operating characteristic. The results suggest differences in the quality and quantity of the data because of segmentation method. The inclusion of linguistic features improved the detection of whole-word repetitions, but not other types of stutters.

**Discussion:**

The findings suggest that event-based segmentation is more suitable for ARS than interval-based segmentation, as it preserves the exact boundaries and types of stutters. The linguistic features provide useful information for separating supra-lexical disfluencies from fluent speech but may not capture the acoustic characteristics of stutters. Future work should explore more robust and diverse features, as well as larger and more representative datasets, for developing effective ARS systems.

## 1 Introduction

Human assessment of stuttering is time consuming and even trained observers give variable scores for the same materials (Kully and Boberg, 1988). If automatic recognition of stuttering (ARS) met acceptable performance standards, these assessments would save time and could standardize score reports. Practical applications other than reducing workload in clinics, include ease of inter-clinic comparisons and making voice-controlled online applications accessible to people who stutter, PWS (Barrett et al., 2022). Given these desirable goals, ARS work began in the late 1990s (Howell et al., 1997a,b). Initial progress was limited because few labs had appropriate training material. Matters improved after the release of the first online audio database of stuttered speech, the University College London Archive of Stuttered Speech (UCLASS) (Howell et al., 2009). UCLASS has time markers indicating where stutters start, and end and the types of stutters are coded (an example of an *‘event-based’* procedure). Each event-based segment varies in duration. Databases that have been established subsequently have segmented speech into fixed length intervals (usually 3 s; 3-s) referred to here as *‘interval-based’* procedures (Lea et al., 2021; Bayerl et al., 2022a). They provide labels for each interval (‘fluent’ or ‘stuttered’ or, in some cases, ‘fluent’ and the specific type of stutter). Whereas fluent intervals are fluent throughout, stuttered intervals with or without symptom-type annotations may not be delimited to these intervals and, when stutters are less than 3-s long, contain some fluent speech and, in some cases, additional stuttered symptoms (Howell et al., 1998). Generally speaking, the fact that intervals are made ambiguous with respect to fluency designation when a fixed duration is imposed onto speech segments, limits overall recognition accuracy of ARS. Surprisingly however, no comparison checks have been made between interval and event-based procedures to verify or disconfirm this prediction.

Additionally, interval-based methods usually report poor performance with respect to whole-word repetitions (WWR) (Lea et al., 2021; Bayerl et al., 2022a). In fact, this might be a correct outcome since there is a wider debate about whether WWR are indeed stutters (Howell, 2010) and because repetition of each constituent word has all phones and these are in their correct positions (implying each word is produced fluently). Whether or not WWR are stutters, it would be difficult to separate them from the same words in fluent speech for procedures that use short-window, acoustic inputs because the segments (events or intervals) may not extend long enough to include any repeated words. In summary, recognition of WWRs and separation of them from fluent speech may be improved if ARS procedures are trained on intervals long enough to include lexical and supra-lexical features (e.g., n-grams for spotting multi-phone repetition representing word and phrase repetition).

Identifying stuttering events may be more appropriate than identifying intervals since stuttering events dominate in clinical and research reports. For example, Stuttering Severity Index (SSI) measures (Riley, 2009) that is partly based on symptoms are always reported in research publications whereas reports that use intervals are rare (Ingham et al., 1993). Additionally, there seems to be little justification as to why a duration of 3-s was chosen as the interval-length other than saving assessment time [see Ingham et al. (1993) for rationale and Howell et al. (1998) for evaluation]. To validate whether 3-s intervals are the preferred length for best ARS model performance, intervals of 2-s and 4-s were also investigated in our study.

The UCLASS database and the Kassel State of Fluency (KSoF) 3-s interval dataset (Bayerl et al., 2022a) were used in the investigation. UCLASS data were also reformatted into the 3-s interval format (intervals of 2-s and 4-s were also computed). UCLASS and KSoF interval data were each used to train and test a shallow (Gaussian support vector machine) and a deep (multi-layered perceptron neural network) machine learning model to establish whether model performance was equivalent for the two datasets. The shallow and deep learning models were then used to determine how model performance was affected by segmentation method for the same (UCLASS) data.

We hypothesized that the distribution of speech types (stutters and fluent speech) should be similar across KSoF, and UCLASS, interval datasets. Also, models trained using these datasets should perform similarly. If these predictions hold, they confirm that UCLASS and KSoF interval data are comparable and validate the subsequent interval-event comparisons made using UCLASS data alone.

Second, performance was compared for models trained on interval-based, or event-based, UCLASS data. It was predicted that models trained using the event-based format would outperform the interval-based models because only the former delimits speech extracts exclusively associated with their fluency types. Area under the curve for the receiver operating characteristic (AUC-ROC) was used as the performance indicator.

Third, the distribution of fluency types for 2-s, 3-s, and 4-s intervals and the effects of using these different-length intervals on model performance were assessed. It was predicted that using shorter interval lengths should lead to a greater proportion of fluent speech intervals relative to disfluent intervals.

Fourth, the model inputs for the 3-s interval and event-based models were switched to investigate whether the features transferred across segmentation formats. Specifically, after training a model on features derived from the 3-s subset of UCLASS, the model was tested on features derived from the event-based subset and *vice-versa*. By switching the feature inputs, this tested whether the parameters learned by one method transferred to the other. This is the first time such a method has been used to investigate whether a trained ARS model is robust to changes in the feature extraction process. A difference was hypothesized in model performance due to this switch, but no direction was hypothesized.

Finally, we compared models with and without language-based features. We hypothesized that the inclusion of language-based features: (i) could lead to better recognition of WWRs, and possibly of fluent speech; (ii) longer intervals should perform better than shorter intervals because of the increased chance that the language-based features identify whole-word repetitions; (iii) using interval data should improve performance over using event data for these models because intervals usually have more scope for including supra-segmental features. Together these experiments should afford a clear and direct comparison of the effects of using the two training-material types on model performance.

## 2 Method

### 2.1 Datasets

UCLASS data are in British English that includes 249 speakers (Howell et al., 2009) of which, audio from 14 speakers with approximately 180 min of valid and labelled speech were used for the current study. The KSoF data are in German from 37 speakers with approximately 230 min of valid and labelled speech (Bayerl et al., 2022a). In both datasets, a hard split was used to keep the speakers in the training, validation and test splits distinct. Hard, separate speaker, splits are key to evaluating models in the ARS field (Bayerl et al., 2022c). As such, the UCLASS data were split into nine speakers for Training, two speakers for Validation and three speakers for Test. For KSoF, 23, six and eight speakers were assigned to Training, Validation and Test, respectively.

#### 2.1.1 The UCLASS dataset

The UCLASS data used has transcriptions at word and syllabic levels aligned against the audio recordings. Annotations also separate fluent speech, prolongations, part-word repetitions (PWR), WWR and blocks. These UCLASS data were segmented at both the event-based and interval-based levels as described below. The breakdown of observations per split varied by segmentation method (Table 1).

**Table 1:** Absolute and relative frequencies of the five classes of speech fluency in the UCLASS event, UCLASS 3-s interval and KSoF 3-s interval subsets.

**Type**	**UCLASS event**		**UCLASS 3-s interval**		**KSoF 3-s interval**	
	**Absolute frequency**	**Relative frequency (%)**	**Absolute frequency**	**Relative frequency (%)**	**Absolute frequency**	**Relative frequency (%)**
Fluent	11,837	82.48	1,228	37.39	1,538	52.91
Prolongation	396	2.76	383	12.69	346	11.90
PWR	469	3.27	733	24.30	339	11.66
WWR	173	1.20	44	1.46	94	3.23
Block	1,476	10.29	729	24.16	590	20.30
Total	14,351	100.00	3,117	100.00	2,907	100.00

##### 2.1.1.1 UCLASS event-based subset

Speech can be annotated at different levels of precision with the event-based method. Here, syllables were the defined event. Applying the event-based scheme to UCLASS yielded 14,351 unique annotations, each of which had a single, valid label.

##### 2.1.1.2 UCLASS interval-based subset

The interval-based format that applies annotations to fixed, 3-s intervals of speech was the main focus in comparisons with event-based methods since this is the only interval length used for ARS to date (Lea et al., 2021; Bayerl et al., 2022a). The continuous UCLASS speech recordings were split automatically into 3-s intervals and their corresponding transcriptions were examined to identify candidate intervals and their type. The interval designation scheme used by Bayerl et al. (2022a) was applied and generated 3,984 intervals. Of these, 3,117 had a single type of stutter or were fluent throughout (valid labels) and 867 were dropped which had either multiple disfluency types, contained interlocutor speech or had no transcription (were silent).

Additionally, interval datasets for 2-s and 4-s were created to investigate the effect of interval length. From the 2-s scheme, 5,985 intervals were extracted. Of these, 3,508 intervals had singular and valid labels. The 4-s scheme yielded 2,982 intervals, of which 2,020 had singular and valid labels. Comparison across the 2-, 3-, and 4-s UCLASS interval subsets is made in section 3.2.2.

#### 2.1.2 The KSoF dataset

The KSoF dataset contains 4,601 3-s intervals of speech of which 2,907 had valid singular labels for fluent speech, prolongation, part-word repetition (PWR), whole word repetition (WWR), and blocks. Here, the data were split into training (*N* = 1,545), validation (*N* = 662), and test (*N* = 700) folds which was the split that Schuller et al. (2022) used. KSoF also has filler (*N* = 390), modified speech (*N* = 1,203), and garbage intervals (*N* = 101). However, since these classes were not available in UCLASS and some are specific to Kassel’s stuttering treatment, these intervals were dropped to allow cross dataset comparisons.^1^ Any intervals where there was more than one type of disfluency within the 3-s interval were dropped in KSoF.

Comparison of the distribution of speech annotations for the UCLASS-Event subset and both Interval sets (Table 1) revealed some marked differences. Fluent speech accounted for >80% of observations in the event subset whereas, the relative frequency of fluent observations in both 3-s interval sets were 37 and 53%. Note that the UCLASS-Event and UCLASS-Interval sets were obtained from the same audio files. The difference in fluency distribution was due to the interval method reducing the percentage of fluent observations by marking whole intervals with disfluent speech as stuttered whereas they often contained some fluent speech. The event-based scheme preserved all instances of fluent speech since stutter labels delimited the exact extent of the disfluent speech. Effectively, the interval method under-samples fluent speech and would lead to interval-based schemes over-estimating stuttering severity. The absolute and relative frequencies of each type of event for the training, validation and test sets are given in a link in section 10.

### 2.2 Feature extraction

Acoustic and linguistic features were extracted to separate stutters from fluent speech. Acoustic features were extracted directly from the audio signal. The linguistic features were derived from a separate speech recognition model’s prediction from the audio signal. Acoustic and linguistic feature sets were generated for all the available audio data.

#### 2.2.1 Acoustic features

**Figure 1:**
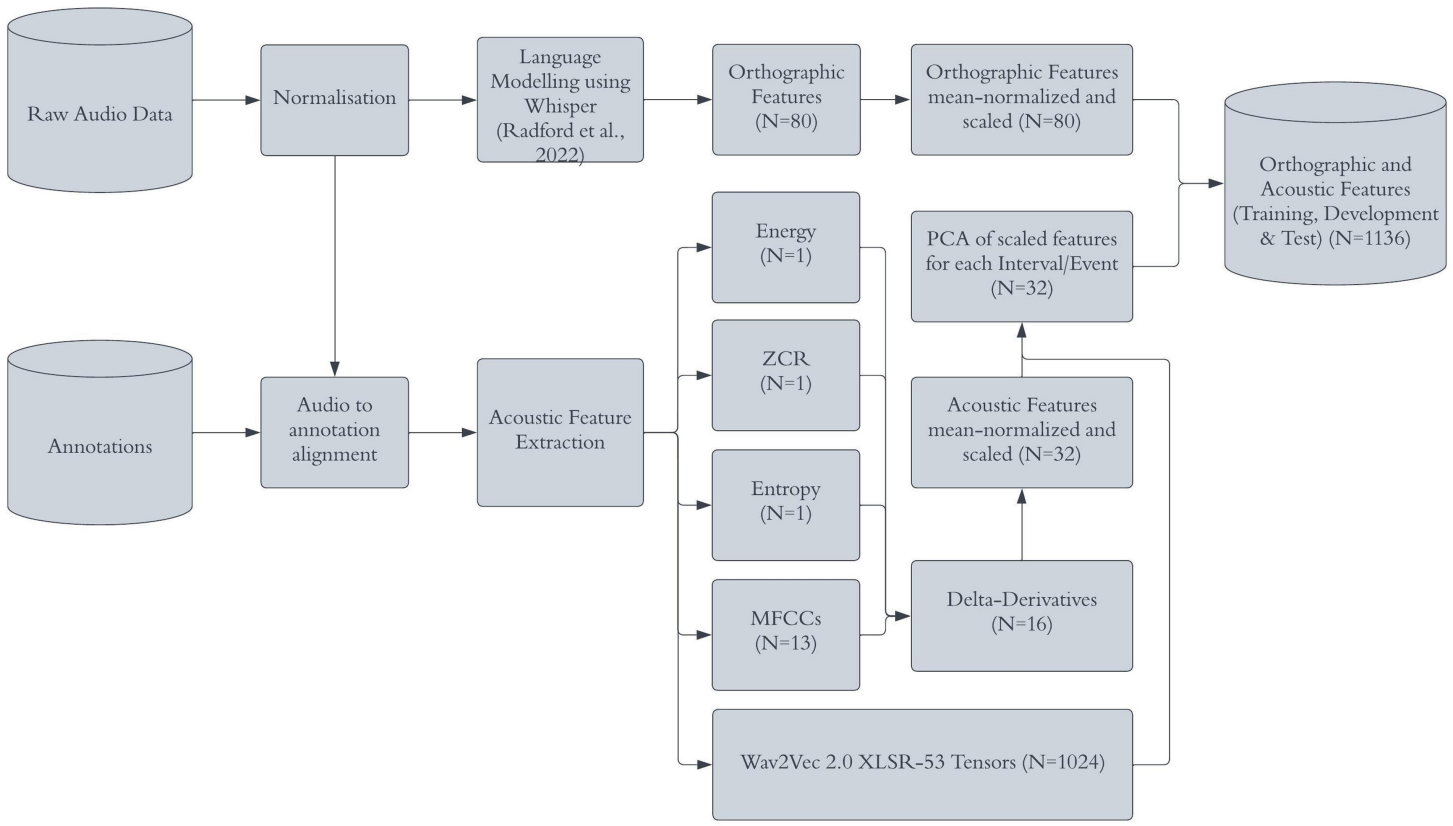
Pipeline for acoustic feature extraction. Reproduced with permission from Barrett (2024).

The acoustic features should provide information concerning how temporal and spectral components change across 2/3/4-s intervals and events. Before acoustic feature extraction was performed, all audio data were normalized such that the oscillogram had maximum and minimum amplitudes in each audio file of +1 dB and –1 dB. For the acoustic set, a set of classical acoustic features were defined. These were: zero-crossing rate, entropy and 13 Mel Frequency Cepstral Coefficients (MFCCs) that were extracted for successive 25 ms time-windows (15 ms overlap). Delta derivatives were calculated across adjacent windows to represent how the features change dynamically across time. Together this resulted in 32 acoustic features per time-frame (Figure 1). These acoustic features are commonly used in the ARS field (Barrett et al., 2022) and pick up on both static (Ifeachor and Jervus, 2002; Tyagi and Wellekens, 2005) and dynamic features of speech (Fredes et al., 2017).

Additionally, a pre-trained deep neural network for representing speech was used to further increase the information presented to the classification models. Here, we used wav2vec 2.0 XLSR-53 (Conneau et al., 2020), as it was trained to represent cross-lingual speech representations from the raw waveform. Note, wav2vec 2.0 XLSR-53 was used for acoustic feature extraction only. For linguistic features a different model, Whisper, was used (Section 2.2.2). The raw waveforms from the data used in this project were inputted to the system, with the resultant tensors of each transformer layer model being used to represent latent aspects of the speech in the signal. This was combined with the classical acoustic features mentioned previously.

The feature matrices were mean-normalized and scaled on the training and validation splits. The resulting feature extraction process produces many features including 1,024 features from the pre-trained network and an additional 32 features from the classic acoustic features. The dynamics of a given feature over the time course of each interval/event was reduced to a singular observation using principal component analysis (Wei, 2019). That is, there was one observation (row of features) for each interval/event. As there were 3,117 intervals in the 3-s UCLASS dataset, its feature set had 3,117 rows.

#### 2.2.2 Linguistic features

The linguistic feature set should provide supra-lexical information that is not readily captured by acoustic features. Stuttered speech contains non-words/syllables that are not included in standard language model vocabularies. Also, disfluent syllables/words/phrases are likely to be infrequent in standard text corpora used to train language models. Furthermore, the audio records in stuttered speech corpora often contain background noise particularly when they are collected in clinical settings. For these reasons, the current state-of-the-art automatic speech recognition model, Whisper, was used (Radford et al., 2023b) first because its architecture can decode speech without a language model, thus enabling it to transcribe both fluent and disfluent speech. Second, it was trained with 680,000 h of audio speech from a wide range of datasets which allows it to be robust against background noise such as those present in stuttered speech audio samples. Finally, its performance on English is reportedly similar to professional human transcribers and it outperformed another state-of-the-art system Wav2Vec2.0 (Conneau et al., 2020) with an improvement of 55% across a range of English datasets.

Whisper comes with multiple pre-trained models. The multilingual model of medium size was chosen. The medium model has 769 million parameters and is capable of transcribing English and German. The medium model performed similarly on English and German with a Word Error Rate (WER) of 4.4 and 6.5%, respectively, on Fleurs (a multilingual dataset).

The respective language identity information (English or German) was provided when transcribing the two datasets.^2^ Given that Whisper was not established for its ability to transcribe stuttered speech including WWRs, we first explored what parameters would encourage a faithful transcription of stuttered speech in a set of small-scale experiments. We found that the default *temperature* parameter influenced its ability to transcribe stuttered speech, especially for WWRs. A model with a temperature of 0 always selected the candidate with the highest probability, and this often failed to generate any repeated syllables/words/phrases in our tests. We therefore experimented with raising the temperature parameter to encourage the model to generate more diverse transcriptions. In our experiments, we generated multiple top-ranked transcription candidates per audio sample. We found that stuttered speech samples were only sometimes faithfully transcribed as one of the candidates, whilst fluent speech samples were transcribed more consistently across transcriptions. We therefore opted to generate multiple possible transcriptions per audio sample following the procedures outlined in the GitHub discussion forum (Radford et al., 2023a). We did this by raising the *temperature* parameter to 0.1 and setting the *best of* parameter to 5 which selected from five independent random samples. Each audio sample was decoded three times, yielding three sets of transcriptions.

The model’s decoding strategy generated transcription chunks (called *segments* in Whisper) which were similar to phrases. The three sets of information returned for each decoded stimulus were: (a) a sequential string of orthographic characters (including spaces and punctuation symbols) for each chunk; (b) the probability of the transcription and the non-speech probability of each decoded chunk; and (c) the timestamps of the acoustic signal that corresponded to each decoded chunk. Sub-sets of Orthographic, Probabilistic and Temporal ARS features were obtained using these respective outputs.

The orthographic features were computed over the entire transcription by concatenating the transcriptions from all chunks. Three types of orthographic features were computed: Sequential lexical n-gram repetition, non-sequential lexical n-gram repetition and non-sequential segmental-n-gram repetition. Sequential lexical n-gram repetition is the number of space-separated-word n-grams which are repeated sequentially. This feature was computed using unigrams to capture word/syllable repetitions, e.g., *das das Buch* “the the book,” and an additional feature used bigrams to capture phrase repetitions, e.g., das Buch das Buch “the book the book.”

Non-sequential lexical n-gram repetition is similar to sequential lexical n-gram repetition, but allows non-sequential repetitions, i.e., not immediately following the n-gram in question, For example, *das Buch nicht das Buch* (“the book no the book”). Two features were computed using unigrams and bigrams, respectively.

Non-sequential segmental n-gram repetition is, in turn, similar to non-sequential lexical n-gram repetition, but applies over characters rather than lexical units. This feature is required because the decoded lexical units had spaces that were not always correctly delimited such that the final instance of prefix repetitions were fragmented. In such cases segmental n-grams can tackle this issue. To avoid detecting repetitions that corresponded to the normal use of repeated syllables/inflectional morphemes in English and German, the repetitions of longer-grams (the length of the orthographic character string minus one) were computed first and. if no repetition was found, then the size of the character n-gram was successively decreased until trigrams were reached. The stopping rule was applied at trigram level to avoid picking up syllable/part-word repetitions. The algorithm stopped immediately at n-grams>3 when repetition was found.

The durations of all the decoded chunks were computed using the timestamps of each chunk. The following summary statistics for temporal features were computed over the durations: the sum, max, min, mean, median, standard deviation, lower quartile (25%), upper quartile (75%), and interquartile range. Two types of probability features were computed: the mean of the probability scores of the transcription and the non-speech probability scores of all decoded chunks.

Each of the above five orthographic features, two probabilistic features and nine temporal ARS features had three values, one from each of the three separately decoded transcriptions. Five summary statistic values (sum, mean, max, min and standard deviations) were computed over each of the three values. The final language-based feature set consisted of 80 feature values [(5 + 2 + 9) * 5 = 80] per audio sample.

As indicated, when applied to continuous speech, event-based segmentation delimits speech types exactly and they vary in duration whereas interval-based segmentation imposes fixed length durations irrespective of the type and extent of speech. Incorporation of language features into the interval-based segments occurs directly when long intervals are used (2-s, 3-s, and 4-s) where interval-length defined the language model’s window. As the best way to provide comparability between event-based models and interval procedures that included language-based features, extracts of speech preceding the event were taken so that events were exactly 2-s, 3-s, or 4-s (as required). Two timeframes around an event were used. One where the *lookback* windows always ended at the end of the event defined for this interval (unlike what occurs in standard interval data). The other was where the event was in the middle of the timeframe. I.e., for a 500 ms event with a 3-s lookback, the linguistic features would be derived from 1.5-s before the end of the event and 1.5-s after the event. Note, the lookback could include other speech classes. Although the acoustic features from an event contain orthogonal information pertaining to the class of that event, the linguistic features contain information that pertains to other classes of speech in some cases. This cross-class information was allowed in the current experiments since this is allowed in standard interval datasets. Possible effects on the resultant models are discussed in section 4.3.

For the interval subsets, the ARS model was run for all interval lengths (2-s, 3-s, and 4-s). For the event-based subset, each event lasted approximately 450 ms on average (Table 2). Hence, the language model would have too short an extract to work with. Consequently, look-backs of 2–3- and 4-s were employed so that the ARS model had equivalent duration to the interval-lengths they were compared with (2-, 3-, and 4-s).

**Table 2:** Estimated mean, standard deviation and quartiles for the length of an event (in ms), split by fluency classes from UCLASS Event subset.

**Class**	**Mean event length (ms)**	**Standard deviation (ms)**	**Lower quartile (ms)**	**Upper quartile (ms)**	**IQR (ms)**
**Fluent**	222	208	102	270	168
**Prolongation**	521	311	313	660	347
**PWR**	763	418	467	980	513
**WWR**	237	155	142	302	160
**Block**	578	467	201	836	635

#### 2.2.3 Summary

**Figure 2:**
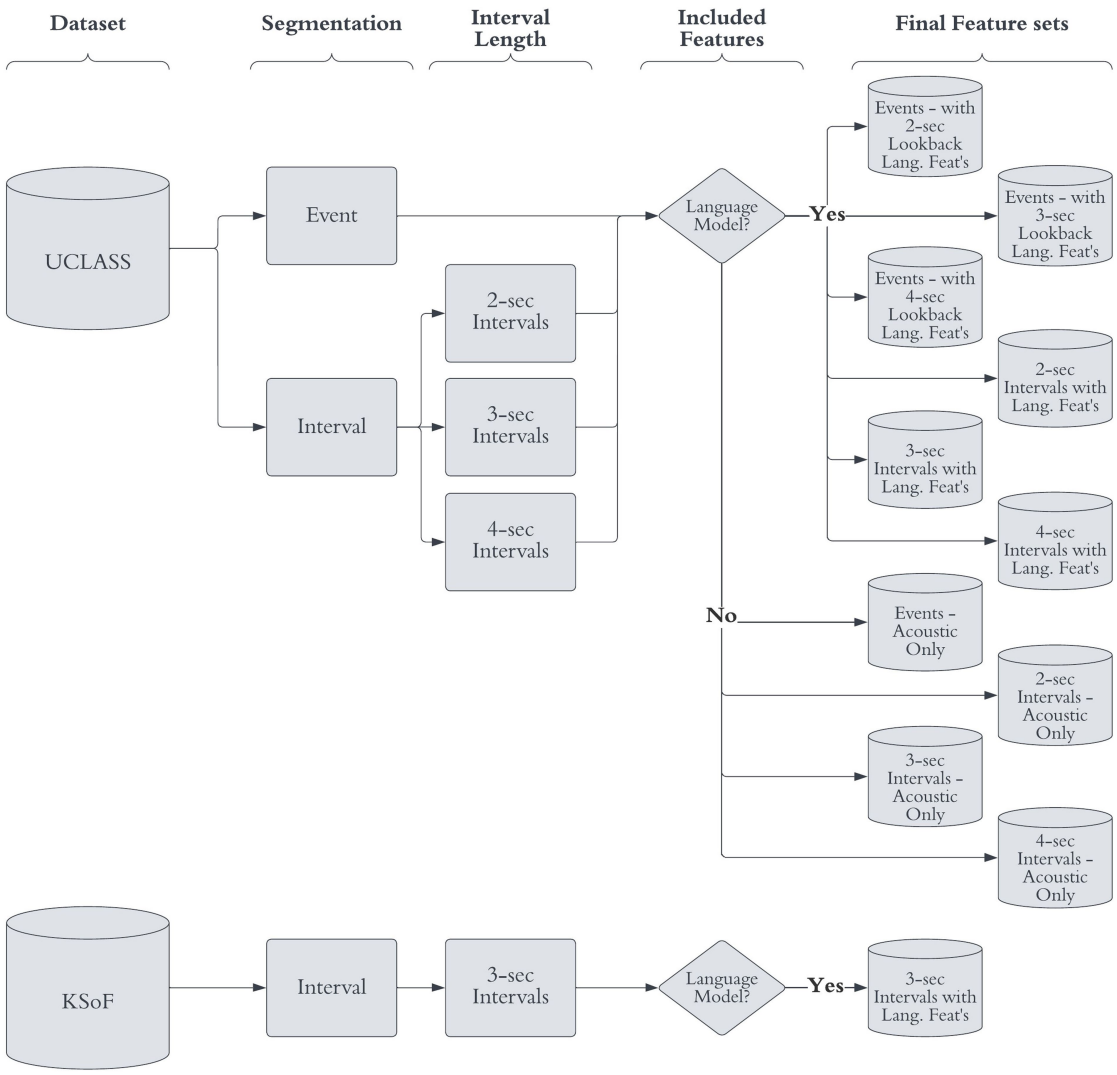
Flow diagram of feature extraction permutation. The final feature-sets used, the original dataset, segmentation method, interval length and included features are given. Reproduced with permission from Barrett (2024).

Thirty-two classic acoustic features were extracted directly from the audio signal. Additionally, the pre-trained acoustic model yielded 1,024 features. The linguistic procedure provided a further 80 features. The two sets of features were concatenated, and z-score scaled (Obaid et al., 2019). This resulted in 11 feature sets (Figure 2) with 1,136 columns and the number of rows equaled the number of intervals/events. The feature sets were then split into training, validation, and test sets, using the same hard, speaker-independent split (Section 2.2).

### 2.3 Metrics

Classification reports are available for each model in the links in section 10. Here, AUC-ROC was the main metric of comparison. This provided an appropriate measure for unbalanced multiclass problems (Jeni et al., 2013) whilst also allowing for simple comparisons. While AUC-ROC provides a reasonable abstracted statistic of model performance, it can mask how the model performs for individual classes. AUC-ROC is used for brevity, but it is recommended to inspect the confusion matrices of all models (see Supplementary materials) for class-level comparisons.

### 2.4 Experimental models

Two types of model are reported in this paper: a Gaussian-kernel SVM (G-SVM) and a multi-layered perceptron neural network (MLP-NN). The Gaussian kernel of the SVM used a penalty term C of 1.15 and gamma varied as a function of the training sets (Equation 1):


(1) $$\gamma =\frac{1}{n\times X_{\mathrm{var}}}$$

Where, n is the number of classes (5) and $X_{\mathrm{var}}$ is the variance of the training set. Predictions were weighted by the class frequencies present in the training set, Equation 2.


(2) $$\omega_{i}=\frac{N}{n\times N_{i}\;}$$

Where $\omega_{i}$ is the weight for the i^th^ class, n was the number of classes (5), N was the total number of observations in the training set and $N_{i}$ was the number of observations in the training set for the ith class.

For the MLP-NN, a sequential deep neural network was constructed with an input layer, five densely connected hidden layers, five drop-out layers and an output layer yielding probabilities for each speech class. The features were input to the first layer with an equal number of nodes. Then, node outputs were propagated through seven densely connected hidden layers, each with a normalization layer with 10% node drop-out. In each of the hidden layers, the outputs were passed through the Rectified Linear Unit activation function (Agarap, 2018), which returned the original input to the function if the input was positive. Finally, outputs from hidden layers were passed through the SoftMax activation function to yield the class probabilities for a given observation. This architecture yielded 819,205 trainable parameters.

The model was trained across 15 epochs with a batch size of 32. Loss was minimized using cross-categorical entropy, which permitted estimation of loss between multi-class probability densities, and was optimized with the solver ‘Adam’, a form of stochastic gradient descent (Kingma and Ba, 2014).

## 3 Results

The field of ARS lacks standards for comparing multiclass models making cross-model comparisons fallible (Barrett et al., 2022; Sheikh et al., 2022). Here, the unweighted AUC-ROC statistic was used as it provides a valid metric for model comparisons as it is virtually unaffected by skewness in datasets and can weight each class of speech equally (Jeni et al., 2013). If a weighted metric was used, it can lead to spuriously high performance due to over-learning fluent speech which is the most frequent class.

### 3.1 Distribution for the datasets

#### 3.1.1 Distribution of the 3-s intervals and event-based subsets

Before report of the model performance on the UCLASS subsets, the differences in overall fluency/disfluency rates between datasets were reviewed. Table 3 gives the absolute and relative frequencies of each class of speech per dataset. Additionally, the ratio of each class of speech relative to fluent speech is given.

**Table 3:** Total and relative frequencies of intervals and events in the KSoF and UCLASS datasets with each datasets ratio of fluent speech to stuttered.

**Sub-set**	**Class**	**Absolute frequency**	**Relative frequency (%)**	**Ratio to fluent speech**
**KSoF|3-s interval (*N* = 2,907)**	**Fluent**	1,538	52.91	1
	**Prolongation**	346	11.90	0.22
	**Part-word repetition**	339	11.66	0.22
	**Whole word repetition**	94	3.23	0.06
	**Block**	590	20.30	0.38
**UCLASS|3-s interval (*N* = 3,117)**	**Fluent**	1,228	39.47	1
	**Prolongation**	383	12.31	0.31
	**Part-word repetition**	733	23.56	0.60
	**Whole word repetition**	44	1.41	0.04
	**Block**	723	23.24	0.59
**UCLASS|Event (*N* = 14,351)**	**Fluent**	11,837	82.48	1
	**Prolongation**	396	2.76	0.03
	**Part-word repetition**	469	3.27	0.04
	**Whole word repetition**	173	1.21	0.01
	**Block**	1,476	10.28	0.12

When segmentation schemes applied to the same data were compared, drastic differences occurred in the relative frequencies of each speech class. In UCLASS-Interval, fluent speech accounted for less than half the labels whereas fluent speech accounted for over 80% of labels in the 3-s Event-based version of UCLASS. A Chi-square test for independence confirmed that the two distributions of speech classes differed significantly ( $\chi_{4}^{2}=3031.80;p<0.001$ ). As discussed in the introduction, this is due to under-sampling the occurrences of fluent intervals. As this paper is approaching stuttering and machine learning from a detection standpoint, fluent speech can be thought of as an absence of stuttering. When removing fluent speech from the distributions we again get a significant difference between UCLASS event and 3-s interval subsets, however with an appreciably smaller statistic ( $\chi_{3}^{2}=305.39;p<0.001$ ).

The distribution of stuttering classes for the 3-s interval types was compared across the KSoF and UCLASS Interval datasets. There was good agreement with respect to relative frequencies of event classes. Both estimated fluent speech to be the most frequent class, although the proportion in KSoF was higher. The higher relative frequency of fluent speech in KSoF was probably due to annotators knowing that a modified speech technique was used by participants (Euler et al., 2009). This would have led to some intervals which would have been categorized as one of the classes of stuttered speech being considered fluent. For example, modified KSoF speech allows intervals that are similar to prolongations to be designated fluent as Bayerl et al. (2022a) noted. Otherwise, the order of stuttering subtype by frequency was usually similar across the 3-s interval datasets. However, KSoF had more part-word repetitions than prolongations whereas the opposite was the case with the UCLASS-Interval subset. This was probably because some prolongation intervals were classified as modified intervals that reduced their incidence in KSoF. A Chi-square test showed that the distributions for the two datasets differed significantly across stuttered and fluent speech ( $\chi_{4}^{2}=207.13;p<0.001)$ . Hence, the hypothesis that the distribution of both the KSoF and UCLASS interval datasets would be homogenous was only partially supported. When fluent speech is dropped, this difference is further reduced ( $\chi_{3}^{2}=98.98;p<0.001$ ). However, the difference between the UCLASS-Event and UCLASS-3 s-Interval distributions ( $\chi_{4}^{2}=3031.80$ ) was still larger than the difference between the UCLASS-3 s-Interval and KSoF-3 s-Interval distributions ( $\chi_{4}^{2}=207.13$ ). This is explored further in section 4.1.

**Figure 3:**
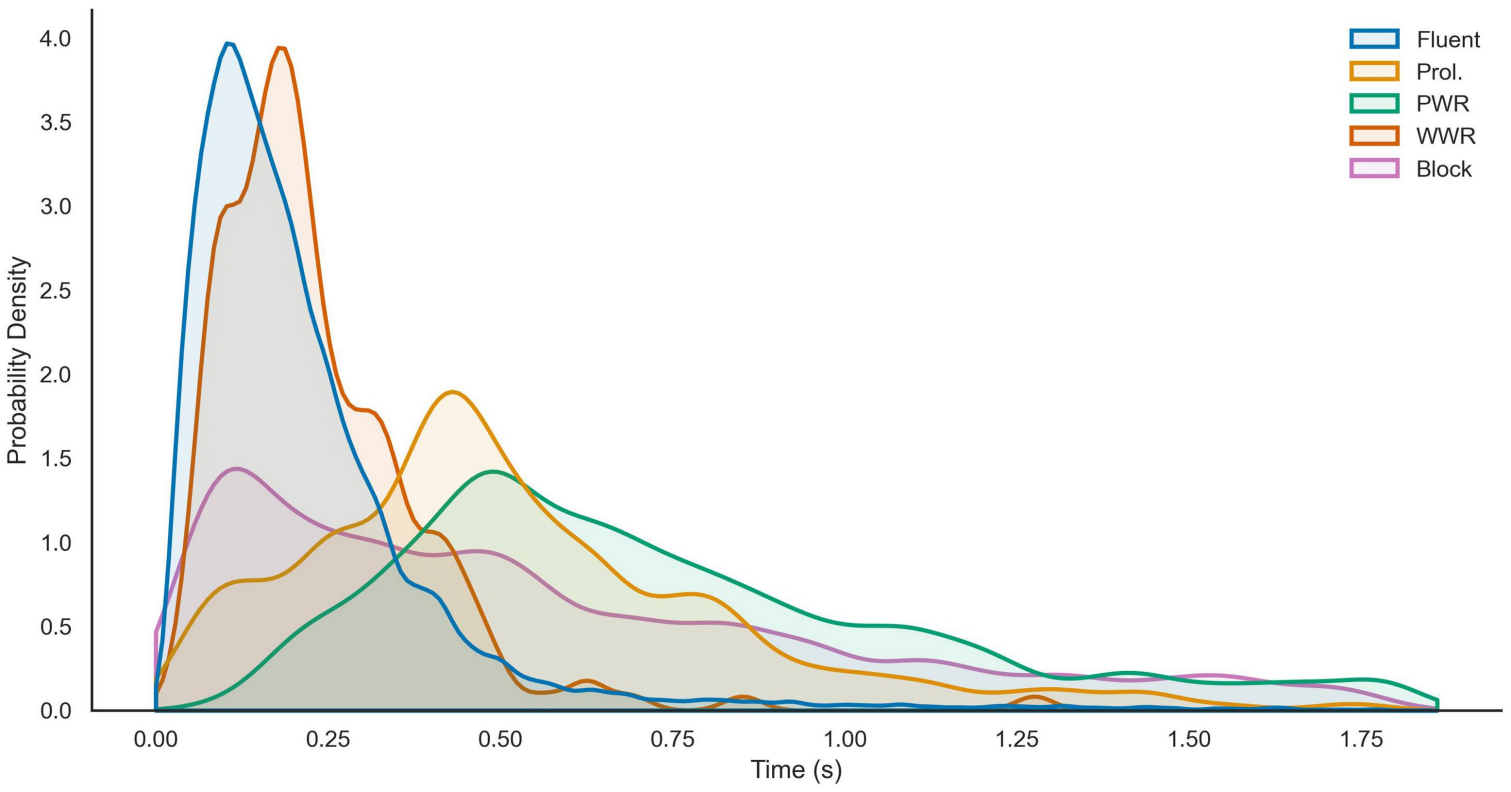
Gaussian kernel density estimates of the relative frequencies of event lengths split by speech class from the UCLASS Event subset. *X*-axis gives event length in seconds and the *Y*-axis shows probability. Reproduced with permission from Barrett (2024).

Unlike the interval subset, where the length of an interval was known *a priori*, the length of events varied. Since the events in the current subset were defined by syllable onsets and offsets, the event length was expected to be approximately 200 ms for fluent speech and 500 ms for disfluent speech (Howell, 2010). Table 2 provides further support for these estimates. This is the first time that the length of stuttered events, split by type, have been reported to our knowledge (Figure 3).

#### 3.1.2 Distribution of fluency types in 2-s, 3-s, and 4-s interval subsets

When UCLASS datasets for different interval-lengths were compared, all had relatively small ratios of fluent to disfluent speech compared to the UCLASS event dataset apart from whole-word repetitions. For interval approaches, a high rate of fluent speech would be expected when shorter time windows (<3-s) were used and a low rate of fluent speech when longer time windows (>3-s) were used. The expected trends in the interval length permutations were confirmed; the shorter the interval, the greater the proportion of fluent speech (Table 4). Prolongations showed much the same relative frequency across the subsets while the proportion of PWR and blocks increased considerably as interval length increased.

**Table 4:** Total and relative frequencies of intervals for 2-, 3-, and 4-s UCLASS interval subsets, split by class.

**Type**	**2-s (*N* = 3,508)**		**3-s (*N* = 3,117)**		**4-s (*N* = 2,020)**	
	**Absolute**	**Relative (%)**	**Absolute**	**Relative (%)**	**Absolute**	**Relative (%)**
**Fluent**	1,532	43.67	1,228	39.40	605	29.95
**Prolongation**	442	12.60	383	12.29	249	12.33
**PWR**	644	18.36	733	23.52	422	20.89
**WWR**	89	2.54	44	1.41	56	2.77
**Block**	801	22.83	729	23.39	688	34.06

#### 3.1.3 Word error rates of the automatic transcriptions

As mentioned, the linguistic features were generated from the outputs of a pre-trained ASR model. While the performance of this model is well documented on reference speech corpora (Radford et al., 2023b), how the model performs with stuttered speech is not known. Here, manual transcripts of the selected UCLASS data were compared against automatically generated transcripts from Whisper. The same model settings were used as defined in section 2.2.2. Whisper’s performance here was evaluated using word-error rate (Equation 3).


(3) $$\mathrm{WER}=\frac{S+D+I}{N\;}$$

Where S is the number of substitutions, D is the number of deletions, I is the number of insertions and N is the number of words in the veridical transcription. In the context of ASR transcriptions, substitutions are where the system replaces the reference word, for example “lose” with phonetically similar hypothesized word, for example “rouse.” Deletions are where a reference word is removed completely from the hypothesis I.e., the “*it*” in the reference “has *it* gone missing” to the hypothesized “has gone missing.” Finally, insertions are hypothesized words that are completely missing from the reference. As in the hypothesis “they wore *many* masks” from the reference “they wore masks.”

Overall, Whisper yielded an average WER of 24.65% across all the UCLASS audio files (Table 5). For comparison, Radford et al. (2023b) reported an average WER of 12.8% across multiple speech corpuses. Stuttered speech presents an almost doubling of WER. This is one of the first investigations of how stuttered speech affects WER of state-of-the-art ASR models. How and why stuttered speech causes such decreases in performance remain unclear. While it is beyond the scope of the current work, this would be well worth further research.

**Table 5:** The word error rate (WER), number of substitutions, deletions, insertions, correct and total words from Whisper (Radford et al., 2023b) split by UCLASS file.

**ID**	**Substitutions**	**Deletions**	**Insertions**	**Correct words**	**Total words**	**WER (%)**
M_0030_16y4m_1	34	9	9	354	406	12.81%
M_0061_16y9m_1	36	30	9	272	347	21.61%
M_0078_16y5m_1	8	18	14	198	238	16.81%
M_0107_07y7m_1	16	29	30	145	220	34.09%
M_0121_11y1m_1	5	29	28	45	107	57.94%
M_0121_15y1m_1	10	26	18	38	92	58.70%
M_0553_10y0m_1	6	22	26	127	181	29.83%
M_0553_11y0m_1	11	14	23	116	164	29.27%
M_1064_47y0m_1	27	90	52	824	993	17.02%
M_1100_28y0m_1	28	88	38	889	1,043	14.77%
M_1101_35y0m_1	36	63	56	470	625	24.80%
M_1103_20y0m_1	35	78	41	555	709	21.72%
M_1104_40y0m_1	28	32	41	602	703	14.37%
M_1105_21y0m_1	63	324	203	765	1,355	43.54%
M_1106_25y0m_1	7	17	16	172	212	18.87%
Total	350	869	604	5,572	7,395	24.65%

### 3.2 Model performance on UCLASS datasets

#### 3.2.1 Event subsets

##### 3.2.1.1 Three second lookback

G-SVM and MLP used the principal components of the acoustic features, the outputs from a pre-trained deep neural net, along with the orthographic features. The ARS model was provided with a maximum of 3-s of audio before the end of the speech event of interest. Where there were less than 3 s of speech available (i.e., within the first 3-s of the audio recording), the length was set to the longest duration available. The G-SVM yielded an average AUC-ROC of 0.83 in test. The MLP-NN performed less well with an AUC-ROC of 0.73. (Table 6 has the full classification report).

**Table 6:** Classification reports of the G-SVM and MLP-NN tested on the UCLASS Event subset.

**Class**	**Gaussian SVM**			**MLP-NN**			**Observations**
	**Precision**	**Recall**	**F1-score**	**Precision**	**Recall**	**F1-score**	
**Fluent**	92.89	75.30	83.18	82.29	17.06	28.83	1822
**Prolongation**	4.03	12.82	6.14	8.82	7.69	8.82	39
**Part-word repetition**	18.60	25.00	21.33	18.52	7.81	10.99	64
**Whole word repetition**	4.17	20.00	6.90	1.56	76.67	3.05	30
**Block**	48.37	70.18	57.27	51.11	58.55	54.57	275
**Accuracy:**			71.39			22.56	2,230
**Unweighted average**	33.61	40.66	34.96	32.26	33.56	21.03	2,230
**Weighted average**	82.52	71.39	75.83	74.77	22.56	30.36	2,230

Given the large imbalance in class frequencies, accuracy should not be used as the sole metric for comparison (Barrett et al., 2022). How performance of these models compared to their interval-based counterparts is reviewed in section 3.3.2.

##### 3.2.1.2 Varying lookback length and window length

Next, 2-s, 3-s, and 4-s lookback lengths were investigated to determine any effects they have on ARS trained on events. When the length of the window was varied with the window ending at the end of the event, there appears to be little effect of varying the duration of the lookback on the model’s ability to classify the current event (Table 7). However, there was a drop off in performance for the NN-MLP when extending the lookback to 4-s (AUC-ROC = 0.69) as opposed to 2-s and 3- lookbacks (both AUC-ROC = 0.73). Additionally, it appears that allowing the linguistic features to represent both the preceding and succeeding speech improved performance with respect to AUC-ROC. This was the case with the NN-MLP models, where performance improved for all window lengths as a result of moving the window to include the preceding and succeeding signal.

**Table 7:** Summary of AUC-ROC scores for each event-based model from the UCLASS data, split by lookback duration (2-, 3-, and 4-s) and context of the language-based features.

**Context**	**2-s (*N* = 2,230)**		**3-s (*N* = 2,230)**		**4-s (*N* = 2,230)**	
	**G-SVM**	**MLP**	**G-SVM**	**MLP**	**G-SVM**	**MLP**
**Before**	0.82	0.73	0.82	0.73	0.82	0.69
**Middle**	0.82	0.75	0.82	0.74	0.82	0.71

‘Before’ is a lookback of N-seconds before the end of the event. Middle is ± $\frac{N}{2}$ seconds around the end of the event.

#### 3.2.2 Interval subsets

As mentioned, the reference interval length was 3-s. The G-SVM and MLP models were trained on the 3-s acoustic and linguistic features. The G-SVM yielded an AUC-ROC of 0.52 at test while the NN-MLP yielded AUC-ROC = 0.54 (Table 8 has the full classification report).

**Table 8:** Classification reports of the G-SVM and MLP-NN tested on the UCLASS 3-s interval subset.

**Class**	**Gaussian SVM**			**MLP-NN**			**Observations**
	**Precision**	**Recall**	**F1-score**	**Precision**	**Recall**	**F1-score**	
**Fluent**	44.68	9.38	15.50	46.83	26.34	33.71	224
**Prolongation**	11.37	57.14	18.97	8.63	28.57	13.26	42
**Part-word repetition**	0.00	0.00	0.00	29.00	24.79	26.73	117
**Whole word repetition**	2.13	20.00	3.84	1.92	10.00	3.23	10
**Block**	23.81	34.48	28.17	26.98	19.54	22.67	84
**Accuracy:**			16.04			24.58	480
**Unweighted Average**	16.40	24.20	13.30	22.67	21.85	19.92	480
**Weighted Average**	26.21	16.04	14.08	34.61	24.58	27.58	480

The hypothesis that event-based data should yield better performance than interval-based data was supported. Performance using AUC-ROC improved when models were trained and tested on data from event-based segmentation rather than from intervals.

The hypothesis that the smaller the interval length, the better the model performance was not supported. Rather the relationship between performance and interval length depended on the type of model used. For NN-MLP models, a quadratic relationship occurred with performance in terms of AUC-ROC peaking when a 3-s interval was used (AUC-ROC = 0.54) and dropping off with smaller (AUC-ROC = 0.48) and longer interval lengths (AUC-ROC = 0.49). In contrast, G-SVM models improved with increased interval length (AUC-ROC 2-s = 0.50; 3-s = 0.50; 4-s = 0.54). However, the variation across interval lengths for both types of model was minor throughout.

#### 3.2.3 Input switching

To further investigate how event- and interval-based inputs influenced how models learned to separate classes of speech, the inputs to the trained models were switched. Thus, models trained and validated on event-based inputs were tested on interval-based inputs and *vice-versa*. This novel method allowed for investigation of a model’s input-invariant properties. The audio data used was the same but the method of segmentation differed. Thus, if performance remained stable, models should be able to separate the classes of stuttering irrespective of segmentation method. When using a NN-MLP architecture, however, switching the input type between intervals and events resulted in models performing equally well, regardless of input (ROC-AUC = 0.54). A G-SVM, model trained on event inputs yielded a greater ROC-AUC (0.57) as compared to the G-SVM trained on intervals and tested on events (ROC-AUC = 0.51). Indeed, the model trained on events outperformed any model trained and tested on intervals (all ROC-AUC in section 3.2.2 < 0.57). This suggests that segmentation method is causal to a machine learning model’s learnt class boundaries. Additionally, G-SVM models trained on event-based data can be used successfully to predict stutters in interval type data.

The hypothesis was made that switching input would yield different responses depending on what the models were trained on. However, it seems that regardless of how the data were segmented during training, if data were used from the other segmentation method, learning performance did not transfer. Therefore, deciding on segmentation *a priori* has lasting effects on their future utility for ARS. Given that event-based procedures are usually employed by speech-language pathologists, SLPs (Riley, 2009), this suggests a preference for training models on event-based data.

#### 3.2.4 Effect of linguistic features

**Figure 4:**
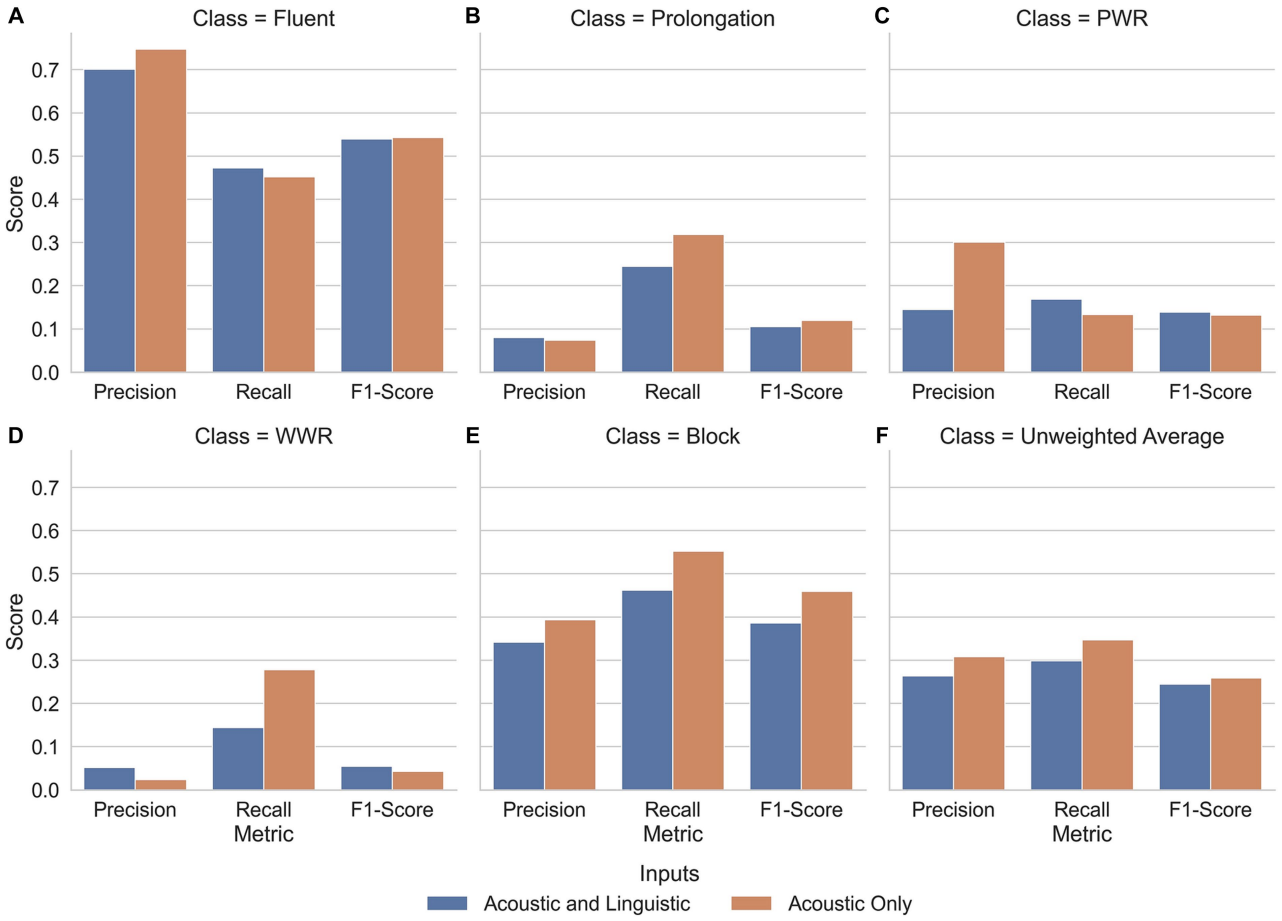
Average precision, recall and F1-score for each class of speech. Inputs to the model are split by inclusion (blue) and exclusion (orange) of linguistic features. Additionally, the unweighted average of each metric are plotted (**F**). Along the *x*-axis of each plot are the metrics precision, recall and F1-Score of each class (**A**. Fluent; **B**. Prolongation; **C**. PWR; **D**. WWR; **E**. Block) as well as the unweighted average across all classes (**F**). These are further split into models which input both acoustic and language features (blue) and models which input only acoustic features (orange). Here, the effect of language features on each class is apparent. Along the *y*-axis, the precision, recall and F1-Scores are measured. The scores are an average of G-SVM and NN-MLP models trained and tested on Event-based inputs with a 2-, 3-, and 4-s lookback. In all stuttering classes, language-based features improved recall and reduced precision as compared to their Acoustic-Only counterpart models. Whereas, in fluent speech, the reverse was true, with recall diminishing and precision improving as a result of language features. Reproduced with permission from Barrett (2024).

As reviewed in the introduction, classification of stutters has usually used acoustic features as input. Here, linguistic features were also used to help separate supra-lexical disfluencies from fluent speech (WWR) as these are reported to be difficult to separate when using acoustic features alone. Performance with the linguistic features has been reported in 3.1.1 and 3.2.2 for events and intervals, respectively. When these features were dropped from the models, using only the acoustic features, similar pattern of results were seen; models trained on events had AUC-ROC_SVM_ = 0.82; AUC-ROC_MLP-NN_ = 0.74 and these outperformed models trained on 3-s intervals (AUC-ROC_SVM_ = 0.54; AUC-ROC_MLP-NN_ = 0.55). For full classification report, visit the link in section 10. Using an AUC-ROC metric, it is not clear whether the linguistic features provided significant benefit to models trained on either Event- or Interval-based data. Indeed, the MLP models trained on events without the linguistic model features performed minorly worse than models with linguistic features, scoring an AUC-ROC of 0.74 as compared to a maximum of 0.75 on events with a 2-s lookback (Table 7). When considering the AUC-ROC, the addition of language-model features provided limited benefit. However, the changes at the class level for precision and recall showed some improvements as a result of language features (Figure 4).

Although the linguistic model features did not systematically improve performance with respect to AUC-ROC, the original purpose was to increase performance with respect to supra-lexical classifications (i.e., WWR). When linguistic features were included, only the disfluent classes of PWR and WWR showed an increase in F1-Score, however the nature of improvement was not the same for the two classes. For PWR, the linguistic features improved the models’ recall while reducing the precision, while, for WWR, the opposite was true. Therefore, a tradeoff emerged between precision and recall, depending on whether PWR or WWR are considered. Another trade-off emerged but, in the identification of fluent speech. Fluent speech followed a similar pattern to PWR, with recall improving through inclusion of linguistic features while precision reduced. The implications of this trade-off are explored further in section 4.3.

**Figure 5:**
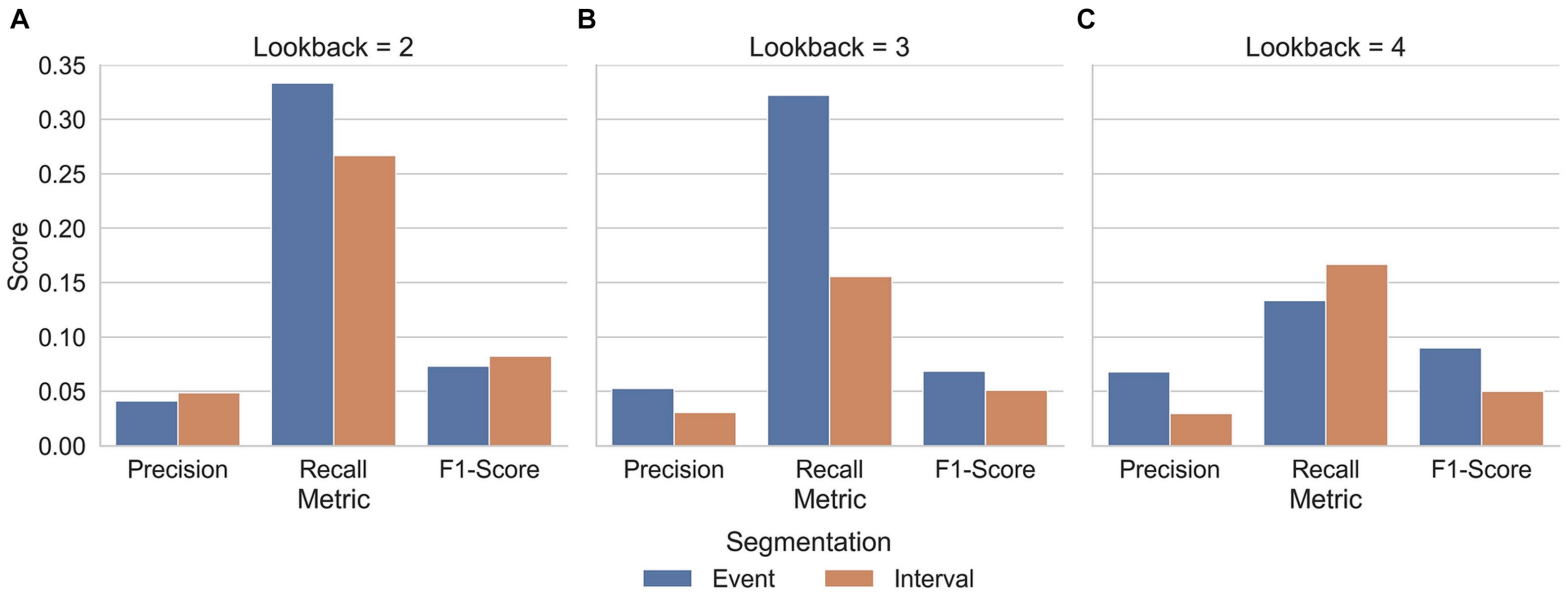
Along the x-axis of each plot are the metrics precision, recall and F1-Score of whole-word repetitions. The y-axis indicates the precision, recall and F1-Scores. Subplots are split by lookback (**A**. 2-s; **B**. 3-s; **C**. 4-s). The scores are an average of G-SVM and NN-MLP models trained and tested on Event- and Interval-based data. Sub-plots are separated by the lookback length given at the top. For Intervals, this is simply the length of the interval. For events, the lookback is the period of time before the end of an event that the linguistic features are derived from. That is, a 3-s lookback means that the linguistic features represent the speech 3-s prior to the end of the stuttered event. Reproduced with permission from Barrett (2024).

When considering WWR’s alone, events yielded better recall in 2-s and 3-s lookbacks (Figure 5). When the lookback was increased to 4-s, however, recall was worse in events than in 4-s intervals. Additionally, precision improved in all event-interval comparisons except when the linguistic features were input with 2-s of speech (Figure 5A).

#### 3.2.5 KSoF interval dataset

The G-SVM yielded an AUC-ROC of 0.55 on the test set. The MLP-NN performed similarly with an AUC-ROC of 0.53 (Table 9 for the full classification report).

**Table 9:** Classification report for Gaussian SVM and MLP-NN models on the KSoF test data.

**Class**	**Gaussian SVM**			**MLP-NN**			**Observations**
	**Precision**	**Recall**	**F1-score**	**Precision**	**Recall**	**F1-score**	
**Fluent**	45.65	23.86	31.13	39.82	33.33	36.29	264
**Prolongation**	22.83	26.36	24.47	27.78	9.10	13.70	110
**Part-word repetition**	22.35	28.79	25.12	18.40	22.73	20.34	132
**Whole word repetition**	5.97	22.22	9.41	2.29	2.78	4.24	18
**Block**	23.23	26.14	24.60	16.13	5.68	8.40	176
**Accuracy:**			25.57			20.43	700
**Unweighted average**	24.01	24.47	23.00	20.88	19.72	16.59	700
**Weighted average**	31.02	25.71	26.84	26.97	20.43	21.90	700

Precision, recall and F1-score were split by each class. Additionally, overall model accuracy, unweighted and weighted average precision, recall and F1-score for each model are reported. Finally, the number of observations for each class is reported.

From AUC-ROC, the G-SVM outperformed the MLP-NN. However, there were substantial differences with respect to sub-class performance. When performance was compared with respect to precision, recall and F1-score, the G-SVM defined the classes of prolongation and part-word repetition better (Table 9), whereas the MLP described fluent speech, whole-word repetition and blocks better in most cases.

It was hypothesized that models trained on KSoF interval data and models trained on UCLASS interval data would perform differently with respect to AUC-ROC. KSoF models yielded AUC-ROC of 0.55 and 0.53 for the G-SVM and MLP-NN, respectively. UCLASS interval models yielded 0.52 for the G-SVM and MLP-NN, respectively. Although the deep learning model provided evidence for the hypothesized result, there does seem to be some non-negligible differences in performance due to the dataset when testing the shallow models.

## 4 Discussion

### 4.1 Summary of results

From the shape of the datasets, interval-based methods yielded a significantly lower proportion of fluent speech (KSoF_Fluent_ = 52.97%; UCLASS|Interval_Fluent_ = 39.47%; UCLASS | Event_Fluent_ = 82.48%). Due to the limited size of the datasets, it was not possible to specify which, if any, stuttering sub-types were over-sampled. The frequency of WWR, however, did not seem to alter drastically by segmentation approach.

**Figure 6:**
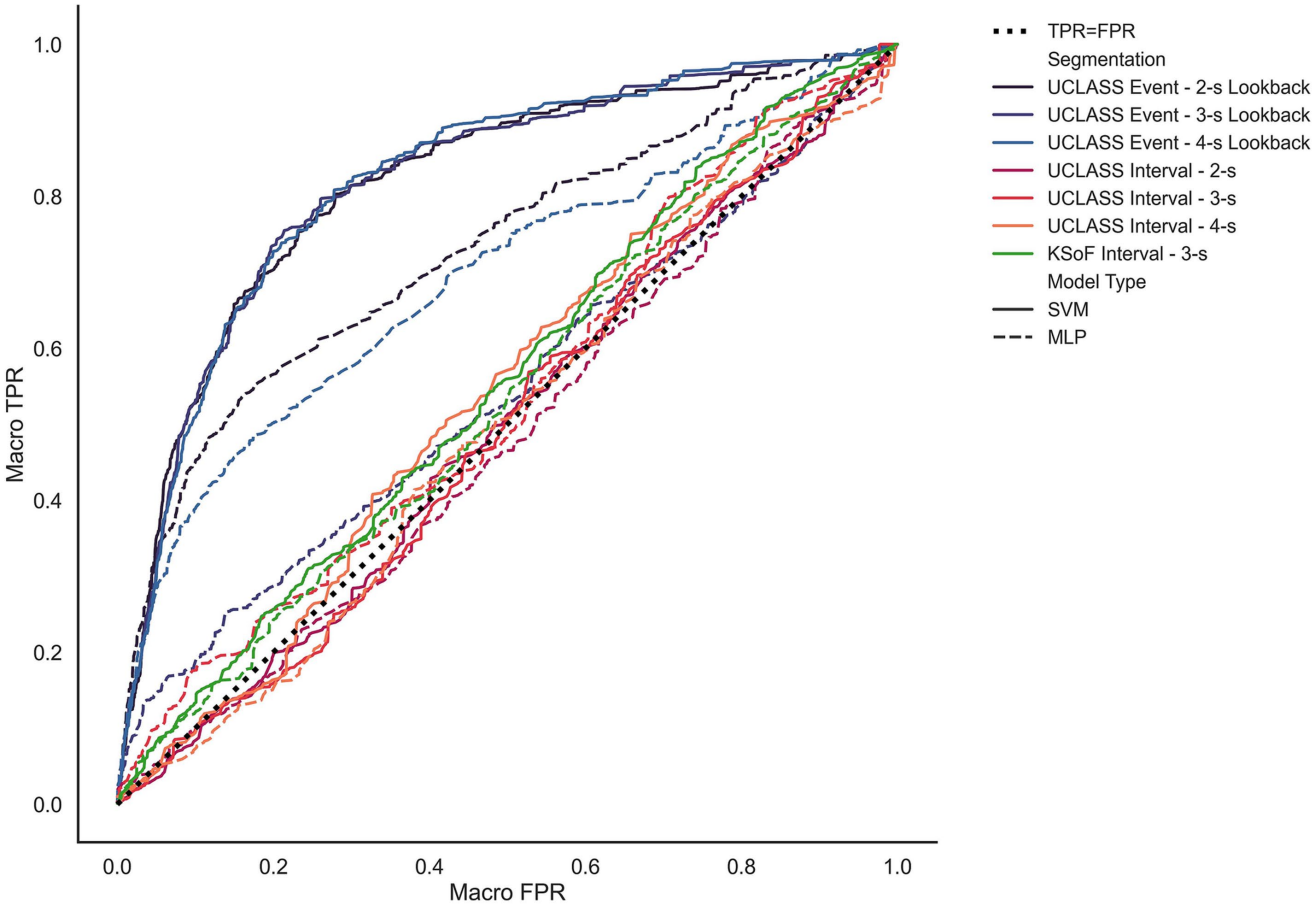
Unweighted average receiver operating characteristics for each model and segmentation method. The *X*-axis measures to unweighted average false positive rate. The *Y*-axis measures the unweighted true positive rate. The black dotted line shows where the true positive rate is equal to the false positive rate (i.e., chance). The solid lines represent performance by the G-SVM and dashed lines represent the MLP-NN. G-SVM models trained on the UCLASS event data yield the largest unweighted AUC-ROC with lookback length inseparable at this level. MLP-NN models on event data performing less well. Finally, all models trained on interval-based inputs vary around the TPR = FPR line. Reproduced with permission from Barrett (2024).

Figure 6 shows the macro-average ROC curves for each model. The models trained and tested on both interval datasets performed poorly with respect to AUC-ROC. Interval models yielded an average macro-AUC-ROC of 0.51. This indicated that these models did not perform above chance when classifying stuttered speech. By contrast, the models trained and tested on event-based data performed reasonably well, with an average macro-AUC-ROC of 0.80.

Models trained on interval data from KSoF and UCLASS showed comparable performance. In both cases, the shallow G-SVM outperformed the deep-learning model on most metrics. It is interesting that the performance on the interval models performed similarly in terms of the ARS problem since they used completely different datasets collected for different purposes. The UCLASS data used here was solely from monologue or conversational speech recorded in the clinic. The KSoF dataset contained speech from monologues in the clinic but, also PWS reading aloud as well as when making phone calls. In KSoF, there were multiple additional sources of variance as compared to the UCLASS Interval subset. As mentioned, the speaking situations varied but also there were more speakers within the KSoF dataset (*N* = 37) than the subset used from UCLASS (N = 14). Additionally, the datasets were in different languages, German and English. The similar performance suggests that the features extracted for the class separation seem to be language independent, at least for those within the Germanic language family. The feature set may extract acoustic features that are universal to stuttered speech which allows fluent and disfluent speech to be separated regardless of the specific language. Future studies should extend examination to other language families to better examine the universality of our acoustic features.

Comparing the current models that used 3-s KSoF data with Schuller et al. (2022) showed that our models performed less well. Schuller et al. (2022) reported only unweighted average recall (UAR), achieving a 37.6 UAR in test using a set of one hundred principal components from a 6,373-feature set. In comparison, using a feature set of 1,136 on the KSoF intervals, the G-SVM yielded a UAR 25.47. Using the UCLASS intervals, a UAR of 24.20 again with a G-SVM. This suggests that performance can be boosted by using further feature dimension reduction techniques. This does not invalidate the conclusion that event-based approaches lead to better machine learning models since the UAR of the UCLASS event-based G-SVM (UAR = 40.66) outperformed Schuller’s reference. Rather, models can be further improved by: (a) Supplying a richer feature set as demonstrated by Schuller et al. (2022); and (b) Using event-based segmentation methods.

The hypothesis that models trained on event-based inputs would outperform interval-based inputs was supported. Both shallow- and deep-learning models trained on events outperformed their interval-based counterparts in terms of AUC-ROC (Tables 7, 9). Indeed, for all aggregate metrics reported (accuracy, weighted and unweighted recall, precision, and F1-Score), the event-based UCLASS models outperformed the interval-based models UCLASS (Tables 6, 8).

### 4.2 Changes in performance due to segmentation approach

Considering the interval- and event-based segmentation method procedures, it was hypothesized that interval-based procedures would limit performance of machine learning models applied to the ARS problem. This hypothesis was confirmed. Interval-based methods led to sub-optimal performances across KSoF and UCLASS datasets compared to interval-based methods.

However, the hypothesis that as the interval length was shortened the performance would increase was not clearly supported. There was some evidence that lengthening the standard interval length from 3-s to 4-s was further detrimental to model performance. In the current study, the minimum interval-length was only reduced to 2-s. As seen in Figure 3, events were closer to 200 (fluent) and 500 ms (disfluent). It may be that further reductions in interval length are necessary to observe the predicted effects on performance.

Additionally, input switching analyses revealed that G-SVM models trained on events and tested on intervals outperformed G-SVMs trained on intervals and tested on events. This suggests that the learnt parameters to identify stuttered speech are somewhat preserved when training on events. Indeed, the parameters learned from events seem to allow for a superior class separation to intervals even when a model trained on events is tested with intervals. As mentioned earlier, event-based approaches allow speech to be delimited such that each observation contains only one class of speech whereas stuttered intervals may contain some, or a majority of, fluent speech. As such, the acoustic features extracted from an event contain no concomitant information from another class, allowing models to learn fine grained differences which are likely to be removed when cross-segment orthogonality is disrupted, as in the interval method. Finally, the input switching analyses also suggest that models trained on events can be successfully used to predict speech fluency on intervals.

### 4.3 Effect of linguistic features

We were not able to find clear evidence that linguistic features increased separation of supra-lexical disfluencies from fluent speech. Despite the addition of features designed to highlight whole-word repetitions, the models overall performed worse when provided with these features. It is unclear why this was the case but, due to the multi-dimensionality of the problem, by increasing the complexity of the inputs to the model, the previously learnt patterns in the acoustic data that help separate sub-lexical disfluencies may become obscured when linguistic features are added. This may explain why linguistic features also reduced the F1 score in speech classes apart from WWR. Another possibility lies with a stopping parameter used to generate the non-sequential segmental n-gram repetition features. The algorithm started with a high n-gram size to find repetitions. The n-gram size decreased if a repetition was not found, whereas it stopped if a repetition was found or if it reached tri-grams to avoid picking up non-lexical repetitions (such as part-word repetitions and prolongation). This tri-gram parameter might be too small to start with and possibly should be increased.

Additionally, linguistic features may have had a detrimental effect on model performance due to possible cross-class correlations within the features. As mentioned, using event-based segmentation the acoustic features represented only the target class. However, the linguistic features incorporated information of up to 4 s before the end of the event. It is feasible, then, that a non-target stutter that precedes the target event influenced the linguistic features. For example, in a 4-s utterance ‘the cat sat sat on the mmmat,’ (target event the prolonged ‘mmm’), there is also the preceding WWR ‘sat.’ The language model would then flag a WWR in the resultant features, leading to contradictory inputs to the ARS model. Future uses of language model features should avoid this issue by ensuring features are relevant to the target event/interval only. Of course, this problem is avoided if event segmentation is employed. We consider that non-orthogonality in the inputs leads to a major limitation in the optimization of ARS modelling. It is proposed that this source of non-orthogonality is a significant factor concerning why the linguistic features did not improve overall performance.

Linguistic features improved the precision of the disfluent classes (including WWR), at the expense of their respective recall. This tradeoff suggests that the quality of the linguistic features has room for improvement. Our small-scale inspection of the ASR transcription suggested that the ASR model would not over-transcribe WWR, that is, the ASR would not transcribe WWR when the signal does not contain WWR (low false positive rate). Future work should inspect the precision and recall of WWR in terms of the ASR transcription. The tradeoff between the recall of the fluent class and the disfluent classes (including WWR) suggests that linguistic features might have helped the model to distinguish between WWR and fluent speech since they cannot be easily distinguished by acoustic features alone.

When considering the quality of the transcripts generated from the ASR system, there is a large error (Table 5). As mentioned, the reference WER of the ASR system used is between 5 and 13% (Radford et al., 2023b). Here, however, an average WER of 24.65% was found with a range of 12.81–58.70%. By far the most frequent type of error made by the ASR system was the deletion of spoken words, reducing the number of transcribed words as compared the number of words actually said by the speaker. Again, deletion here is the complete removal of a word present in a reference transcript in the ASR’s hypothesized transcript. I.e., the “it” in the reference “has it gone missing” to the hypothesized “has gone missing.” Note, this is the number of errors by the ASR system and not the number of errors (stutters) by the speaker. This may be due to stuttered speech being ignored by the ASR system, resulting in a loss of words transcribed. However, a dedicated analysis is required to confirm this hypothesis which is beyond the scope of the current paper. Additionally, deletions might result from the ASR system removing repetitions. Despite the current paper’s attempt to reduce this through adjustment of the hyper-parameters (See section 2.2.2). This not only increases the estimated WER of the system but also removes information of interest for the current purposes. The relatively poor quality of the transcriptions may, therefore, contribute to the current linguistic features’ limited effect on model performance. This poor performance of ASR on stuttered speech was also found in (Thomas et al., 2023) which examined the potential for enhancing automatic cognitive decline detection (ACDD) systems through the automatic extraction of disfluency features using ASR systems. The accuracy of ACDD systems was much lower (78.4%) when trained on automatic disfluency annotations than when trained on manual annotations (88.8%).

When model type and segmentation method were combined and the overall difference between models with and without linguistic features was compared (Figure 5) a consistent trade-off between precision and recall emerged. In all stuttering sub-classes, recall improved and precision worsened with the inclusion of features from a language model whereas in fluent speech, the opposite was true; recall worsened and precision improved.

Precision is the ratio of true positive predictions to all positive predictions. Hence, in the fluent speech class precision is the percentage of correct predictions for fluent speech out of all a model’s predictions of fluent speech. Recall is the ratio of true positive predictions to all instances of the chosen class. In fluent speech, it is the percentage of correctly predicted cases of fluent speech out of all fluent observations.

Therefore, it seems as though language features improved the representation of stuttering classes at a population level. However, the features also lowered the confidence in an individual prediction being true. In contrast, for fluent speech, language features resulted in an increase in confidence of a prediction being true.

The precision-recall trade-off leads to a decision on the aims of the ARS model. In other fields of machine learning which focus on symptom detection, such as cancer, a high recall is preferred over a high precision since the cost of missing a case of cancer is greater than a false positive. In the field of ARS, however, it is not clear whether precision is preferred over recall or *vice-versa*. For Speech and Language Pathologists who may review the predictions, an emphasis on recall may be optimal since wrong predictions can be resolved later.

As Dinkar et al. (2023) noted, a language model’s abstraction from audio input to textual output may result in critical loss of the information which makes the speech stuttered. State-of-the-art language models are often trained using highly fluent materials which are unrealistic in real world scenarios and indeed the audio used in the current work. A large increase in WER was reported here for transcriptions from PWS’s speech. This may explain why the linguistic features were of limited benefit to the ARS models. The linguistic features often provided inaccurate information about the represented speech, reduce class separation. For linguistic features to be better utilized for the ARS classification problem, the ASR systems themselves need to be improved for stuttered speech. Work by Rohanian and Hough (2021) highlighted how the ASR outputs can be modified to better capture certain types of disfluent inputs. However, this was limited to fillers in Rohanian and Hough (2021)work. Further work is required to investigate: (a) which stutters are vulnerable to reduction in an ASR’s outputs; and (b) how to improve the ASR’s outputs for the aims of stuttering detection.

### 4.4 On event-based approach

The current study presented consistent evidence that the event-based procedure for segmenting stuttered speech allowed models to better classify stuttered and fluent speech than the interval procedure. Regardless of whether the models were shallow or deep, whether language features were included or not and irrespective of length of interval, all event-based models outperformed all interval-based models in AUC-ROC (Tables 7, 9), amongst other metrics. Therefore, it is highly recommended for future research to employ event-based data to train ARS models.

Beyond the practical implications, the results also highlight the importance of class orthogonality in training. A key difference in the features provided to the models by event- and interval-based schemes is the level of cross-class orthogonality. Although the interval-based scheme resulted in no cross-stutter correlation (no intervals contained more than one class of stutter), there was a significant level of fluent-stutter correlation. From the novel analyses on event lengths (Section 3.1.1.), a prolonged syllable is on average 521 ms. Given a 3-s interval labelled as prolonged, with a single prolonged syllable, the expected proportion of audio which pertains to the labelled class is only a sixth of the audio used for feature extraction. The other five-sixths audio provide information of non-target classes (fluent speech, silence, noise, etc.).

However, the event-based procedure is not without its limitations. First, event-based approaches require labelling events rather than intervals. This is time-consuming, with limited opportunity for automation. Syllabic levels of transcription and annotation, as used here, often require expertise in linguistics for reliable markers within the signal to be inserted. Also, unlike interval-based labelling, label permutation or over−/under-sampling methods are not feasible.

Second, as shown in 3.1.1, event-based segmentation resulted in a large class imbalance. The classes of interest (stutters) were dramatically skewed by the predominance of the fluent class. Although this is representative of fluency rates in PWS, this does lead to possible limits and pitfalls for machine learning approaches (Gosain and Sardana, 2017). Given this class imbalance, it is more surprising that event-based models outperformed interval-based models uniformly, as the latter allow for a more balanced dataset. From Table 3, the relative frequency of blocks in the event-based segmentation (10.28) was less than half the relative frequency of blocks in the 3-s interval-based segmentation (23.24%). Yet, the event-based models’ ability to represent blocks outperform the interval-based models in every reported metric (Tables 6, 8). Again, event-based data provides superior materials for training ARS models. However, there may still be detrimental effects of this class imbalance. The same 3-s interval models outperformed the event-based models in certain metrics on prolongations and part-word repetitions. Therefore, when using event-based approaches, future research may benefit from using methods to counteract this class imbalance.

Third, the event-based approach assumes *a priori* knowledge of event onset and offset times. When given an unlabeled, purely continuous audio stream, a separate event onset-offset model would be required. This contrasts with the interval-based approach where the audio stream is automatically ‘chunked’ into the prior set time intervals. Also, how one segments events in speech in an online, real-time approach is a further limitation. In the interval-based scheme this problem is trivial. Buffer the Input by the length of the pre-set time window (e.g., 3-s), perform feature extraction and reduction over the signal in this timeframe and feed the resultant features to the model. As discussed above, this may inherently limit the speed of predictions of a model using the interval-based scheme since there is a preset buffer, in our case, 3 s.

Overall, the event-based procedure for speech segmentation provided the best training materials for ARS models.

### 4.5 On interval-based approach

There are several aspects of the interval-based approach that could be automated where the event-based one cannot. For example, the time duration of an interval is preset. Hence, extracting intervals from a file is easy to process whereas, events must usually be done manually. As traditional ASR models can automate word/phonemic boundary locations in speech, the events could feasibly be automated at this stage also. In a similar vein, the annotator does not need to be trained on separating linguistic components of speech (i.e., syllables, phonemes, etc.) in the interval-based method. This is another stage at which the event-based procedure is more time consuming and costly. However, an interval-based approach cannot ensure that an interval contains only one type of stuttering. Therefore, unless using a multi-label system, the interval approach is fallible to data loss where the event is not. Bayerl et al. (2022b) used a multi-label approach on interval-based data with positive results. Models were able to incorporate this more complex multi-label information without detriment to model performance relative to single-label methods as in Lea et al. (2021) and Bayerl et al. (2022a). Therefore, if using an interval-based dataset, a multi-label approach should be used to limit data drop-out.

Finally, given the inputs to an interval-based model are temporally inflexible, the interval procedure is highly applicable. In the event procedure, events would first need to be separated out in online speech classification, requiring a phoneme recognizer as an initial layer to the model. Whereas an interval method simply makes predictions about the interval provided. For example, a 3-s interval model would be able to make predictions on any 3-s input of audio signal. This does, however, also lead to a critique of the interval method in that the classification speed is, at minimum, the same lag as the interval speed. It therefore seems incompatible with real-time uses where latency is critical.

### 4.6 On whole word repetition

In both the current paper and a baseline model for kSoF (Schuller et al., 2022), WWR was the most difficult class of speech to recognize. Unlike blocks or prolongations, for example, WWR have no within-word disfluency. Rather, the perceived disfluency is only identified at the word or phrase level. For instance, the prolongation in “The cat ssssat on the mat” occurs on the “s,” alone. Whereas, in the phrase repetition “The cat sat on the on the mat,” the disfluency occurs across the two words “on the.” Given that the models presented here were mainly based on acoustic features with no language model or decoder-encoder components, would WWRs be separable from, for example, fluent speech? It is proposed (a) that WWR are not separable at an acoustic level and (b) they should not be included in the same roster as sub-segmental disfluencies.

Point (a) is supported by the spread of model predictions when an instance of a WWR is input to the model. In Schuller et al.’s (2022) model and the 3-s interval MLP-NN model, WWRs were predominantly assigned to the ‘Fluent’ speech class. In the Gaussian SVM, the ‘Fluent’ class was the second-best predicted class for true instances of WWRs after Fillers.

It was hypothesized that the inclusion of language features would increase class separation for WWR. There is limited evidence for this hypothesis. Recall rate improved across all stuttering classes after inclusion of language features. Although language features were detrimental to precision, the theoretical motivation remains clear; if WWR cannot be separated from fluent speech at an acoustic level, information at the supra-segmental/lexical level is required. Continued exploration of features such as phone and word-level n-grams is suggested with special attention to sequence of words. Further investigation with more complex language models may help solve this class inseparability.

### 4.7 Clinical implications

The human assessment of stuttering, a significant bottleneck for clinical work, is costly in terms of valuable clinical time and often yields variable assessment outcomes (Kully and Boberg, 1988). Automated procedures have promised to lighten workloads (Howell et al., 1997b; Barrett et al., 2022), but they have yet to be implemented in clinical practice.

Despite 25 years of research into automatic stuttering detection and labelling, a significant trade-off remains between model flexibility and model performance. Models are either highly specific to a task within stuttering recognition and yield adequate performance for application in a clinic (Mahesha and Vinod, 2016), or they are flexible enough to better handle the complex nature of stuttering and its classification but do not meet the necessary standards for use in clinical settings. For example, Mahesha and Vinod (2016) present a Gaussian Mixture model with an approximate 95% accuracy. However, the model is only able to classify repetitions (it is unclear whether this includes PWR, WWR or both), prolongations, and interruptions. The models presented here, as well as those presented in Lea et al. (2021) and Mishra et al. (2021), among others (See Barrett et al., 2022 for review), all perform with less than 95% accuracy.^3^ However, some works, such as that by Gupta et al. (2020), which achieve more than 95% accuracy, are trending towards a level of performance where use in a clinic should be considered. It is unclear whether the model was provided with an event- or interval-based segmentation scheme.

The study provides compelling evidence that employing event-based procedures enhances the capacity of machine learning models to address the ARS problem in comparison to interval-based procedures. This observation is congruent with common human assessment practices for stuttering (Riley, 2009), which frequently utilize event-based metrics like the percentage of syllables stuttered. Models generated through event-based procedures offer predictions based on events and seamlessly align with prevailing clinical practices, presenting an avenue for not only partially automating stuttering severity assessment but also achieving full automation. While the current models provide important insights for ARS research, they are not suitable for use in clinical scenarios ‘out-of-the-box.’ As mentioned earlier, the performance levels do not meet the necessary standards. This is demonstrated by a comparison of the true and predicted cases of stuttering in the test (see the Supplementary materials for confusion matrices). For example, the 3-s event-based G-SVM with linguistic features included (described in section 3.2.1.1) yielded a set of predictions (see Supplementary Data Sheet 10) which significantly changed the shape of the speech fluency distribution. Event-based segmentation types led to an approximate distribution of 83% fluent, 3% prolongation, 3% PWR, 1% WWR, and 10% block/break. This approximates the true distribution in the test set. However, if one were to implement automated labelling using the aforementioned model (arguably the best presented here), the shape of speech fluency changes drastically: 46% fluent, 12% prolongation, 3% PWR, 24% WWR, and 15% block/break. Clearly, the models presented are for research purposes only and not for use in the clinic.

While in-clinic work with ARS models has yet to take place, the current work contributes to a growing field providing proof-of-concept evidence that ML models could improve workflows in clinical assessments of stuttering. The current work strongly supports the use of event-based segmentation in the preparation of data for ARS models. Additionally, this form of segmentation fits well with commonly used stuttering assessments (Riley, 2009). Future work should seek to compare how partial and full automation of stuttering assessment performs in comparison to the current standard (no automation). Research should consider the trade-off between the time taken for the assessment and the error imparted due to automation.

## 5 Conclusion

The current work investigated methods of speech segmentation for machine learning classification. The two main methods of speech segmentation for stuttering classification have been employed: interval- and event-based. While interval-based methods are time and cost effective, event-based methods yield far superior models with less data. This is particularly pertinent given the lack of openly-available stuttering event data currently available (Barrett et al., 2022). Further research could make use of the additional interval databases (Lea et al., 2021) to provide further power to the current study’s findings.^4^ It is therefore highly recommended that future research uses event-based segmentation methods to build stuttering classifier models. Software to add annotations about stuttering events (onsets, offsets, and stuttering type) to continuous audio files has been provided in Howell and Huckvale (2004).

## Data availability statement

The datasets generated and analyzed for this study can be found in the paper’s Open Science Framework Page [https://www.doi.org/10.17605/OSF.IO/29K7Q] and KSoF [https://doi.org/10.5281/zenodo.6801844]. The features, saved instances of the models used and code used to generate these objects are available in OSF.

## Author contributions

LB: conceptualization, investigation, methodology, software, visualization, data curation, writing- original draft preparation. KT: conceptualization, methodology, software, data curation, and writing. PH: conceptualization, supervision, writing- reviewing and editing.

## Footnotes

^1^Since certain classes of speech were dropped from KSoF to allow comparison with UCLASS, the number of observations in each split differed from that reported in (Schuller et al., 2022).

^2^Note that we did not rely on Whisper’s ability to automatically identify the language from speech because its reported performance is not competitive, and it was not an objective of the current study.

^3^95% accuracy is chosen as a threshold as we consider that for adequate use in the clinic. In that, the probability that a predicted dysfluency is not actually present for a given prediction should be at least 0.05 or lower. Further work should seek to establish a set of thresholds across accuracy, precision and recall – amongst other metrics – both theoretically and empirically to guide application to clinical settings. While state-of-the-art performance of ARS models can vary freely within research, translation to in-clinic practice requires a separate set of baseline standards.

^4^We would like to acknowledge the reviewers for their suggestions and recommendations on including all datasets available in the future.
