# Corrigendum: Comparison of performance of automatic recognizers for stutters in speech trained with event or interval markers

**DOI:** 10.3389/fpsyg.2024.1507956

**Published:** 2024-11-06

**Authors:** Liam Barrett, Kevin Tang, Peter Howell

**Affiliations:** ^1^Department of Experimental Psychology, University College London, London, United Kingdom; ^2^Department of English Language and Linguistics, Institute of English and American Studies, Faculty of Arts and Humanities, Heinrich Heine University Düsseldorf, Düsseldorf, Germany; ^3^Department of Linguistics, University of Florida, Gainesville, FL, United States

**Keywords:** stuttering, speech pathology, automatic speech recognition, machine learning, computational paralinguistics, language diversity, language model, whisper

In the published article, there was an error in Figure 3. The figure was supposed to include data for a type of stuttered speech (Prolongations or “Prol.”). The corrected Figure 3 and its caption appear below.

**Figure 3:**
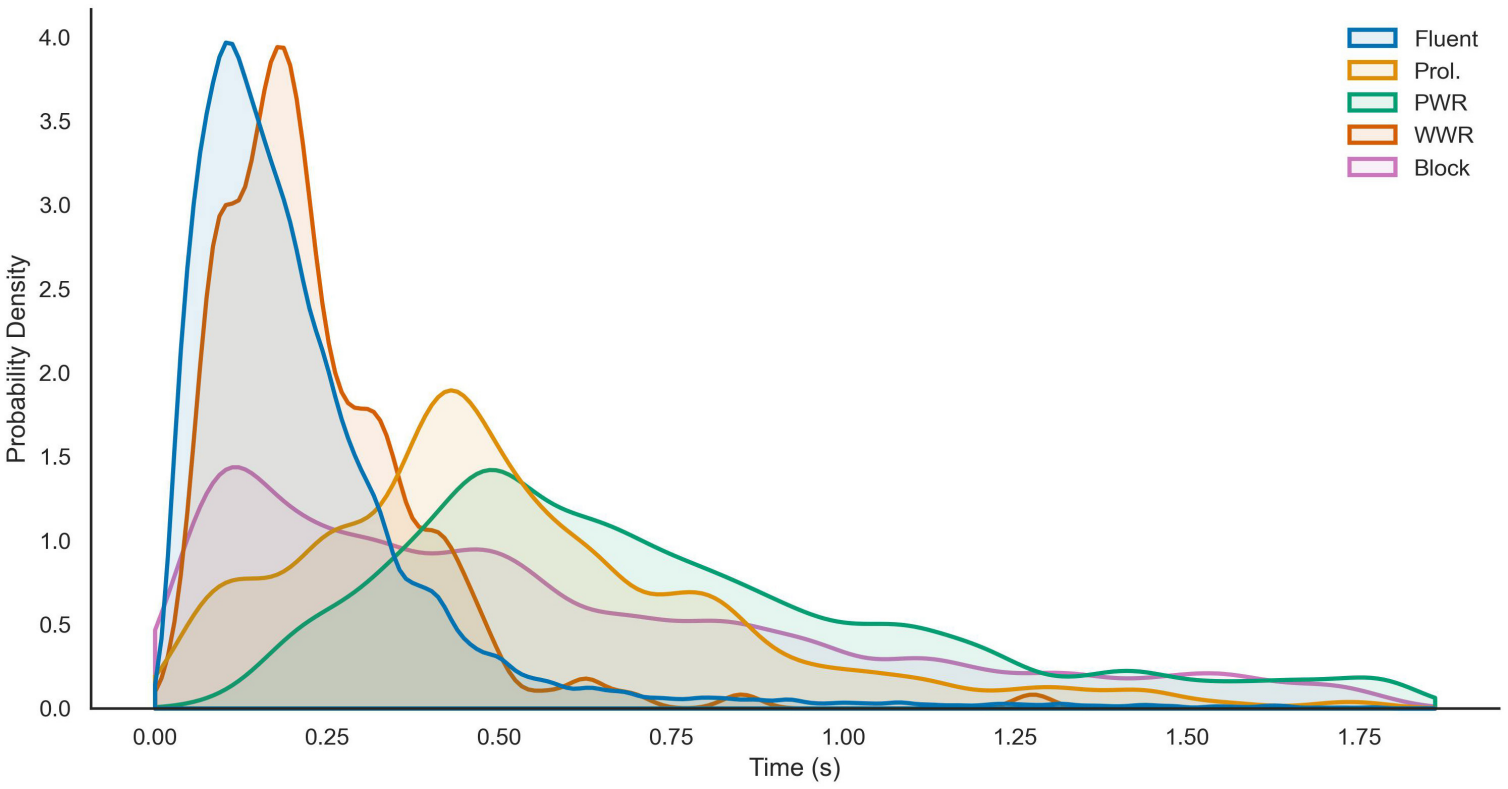
Gaussian kernel density estimates of the relative frequencies of event lengths split by speech class from the UCLASS Event subset. *X*-axis gives event length in seconds and the *Y*-axis shows probability. Reproduced with permission from Barrett (2024).

The authors apologize for this error and state that this does not change the scientific conclusions of the article in any way. The original article has been updated.

